# Supplementary material for: Individual, Family, and Social Factors Associated with Gestational Weight Gain in Adolescents: A Scoping Review
Source: Nutrients. 2023 Mar 22;15(6):1530. doi: 10.3390/nu15061530 (PMC10058126; doi:10.3390/nu15061530)
Supplement: Supplementary file 1 [file nutrients-15-01530-s001.zip › nutrients-2246371-supplementary.pdf]

Table S1: Search strategy to identify articles indexed in the different databases.

Search num	Search terms
1	*"Adolescent pregnancy"* [Title/Abstract] OR *"Teenage pregnancy"* [Title/Abstract] OR "Young pregnancy" [Title/Abstract]
2	" Gestational weight gain"[Mesh]
3	1 AND 2
4	"*Inheritable*" [Title/Abstract] OR "*Genetic variant*" [Title/Abstract] OR "*Polymorphism*" [Title/Abstract]
5	"*Pre-gestational weight"* [Title/Abstract] OR "*Pregestational weight"* [Title/Abstract] AND "*Body fat*" [Title/Abstract] OR "*Pregestational BMI*" [Title/Abstract] OR "*Pre-gestational BMI*" [Title/Abstract] OR "*Pre-pregnancy BMI"* [Title/Abstract], OR "*Underweight*" [Title/Abstract], OR "*Overweight*" [Title/Abstract], OR "*Obese*" [Title/Abstract].
6	"*Diet*" [Title/Abstract] OR *"Nutrition"* [Title/Abstract] OR *"Nutritional status"* [Title/Abstract] OR *"Nutritional intake"* [Title/Abstract] OR "*Physical activity*" OR "Exercise" [Title/Abstract] OR "*Depression*" [Title/Abstract] OR [Title/Abstract] "*Emotional factors*" OR "*Stress level*" [Title/Abstract], OR *"Anxiety"* [Title/Abstract] OR *"Maternal mental health"* [Title/Abstract].
7	"*Family*" [Title/Abstract] OR "*Family support*" [Title/Abstract], OR "*Maternal support*" [Title/Abstract], OR "*Paternal support*" [Title/Abstract], OR "*Partner support*" [Title/Abstract].
8	*"Health care"* [Title/Abstract] OR *"Accessibility*" [Title/Abstract] OR "*Environmental*" [Title/Abstract] OR *"Culture"* [Title/Abstract] OR *"Beliefs"* [Title/Abstract] OR *"Knowledge"* [Title/Abstract] OR *"Economic system"* [Title/Abstract] OR *"Ethnicity"* [Title/Abstract], OR "*Affordability of care*" [Title/Abstract] OR "*Maternity care model*" [Title/Abstract] OR "*Continuity of care*" [Title/Abstract] OR "*Acceptability of care*" [Title/Abstract]
9	3 AND 4
10	3 AND 5
11	3 AND 6
12	3 AND 7
13	3 AND 8

Table S2: Evaluation of the quality of the information

Article	1	2	3	4	5	6	7	8	9	10	11	12	13
Was the research question or objective in this paper clearly stated?	1	1	1	1	1	1	1	1	1	1	1	1	1
Was the study population clearly specified and defined?	1	1	1	1	1	1	1	0	0	0	1	1	1
Was the participation rate of eligible persons at least 50%?	1	1	1	1	1	1	1	1	1	1	1	1	1
Were all the subjects selected or recruited from the same or similar populations (including the same time period)? Were inclusion and	1	0	1	1	1	1	1	1	0	0	1	1	1

exclusion criteria for being in the study prespecified and applied uniformly to all													
Was a sample size justification, power description, or variance and effect estimates provided?	0	0	0	0	0	0	0	0	0	0	1	0	0
For the analyses in this paper, were the exposure(s) of interest measured prior to the outcome(s) being measured?	1	1	1	1	1	1	1	1	1	1	1	1	1
Was the timeframe sufficient so that one could reasonably expect to see an association between exposure and outcome if it existed?	1	1	1	1	1	1	1	1	1	1	1	1	1
For exposures that can vary in amount or level, did the study examine different levels of the exposure as related to the outcome (e.g., categories of exposure, or exposure measured as continuous variable)?	1	0	0	1	1	1	1	1	1	1	1	1	1
Were the exposure measures (independent variables) clearly defined, valid, reliable, and implemented consistently across all study participants?	1	1	1	1	1	1	1	1	1	1	1	1	1
Was the exposure(s) assessed more than once over time?	1	1	1	1	1	1	1	1	1	1	1	1	1
Were the outcome measures (dependent variables) clearly defined, valid, reliable, and implemented consistently across all study participants?	1	1	1	1	1	1	1	1	1	0	1	1	1
Were the outcome assessors blinded to the exposure status of participants?	1	1	1	1	1	1	1	1	0	0	1	1	1
Was loss to follow-up after baseline 20% or less?	1	0	1	0	1	1	0	1	0	0	1	1	1
Were key potential confounding variables measured and adjusted statistically for their impact on the relationship between exposure(s) and outcome(s)?	0	0	0	1	1	1	1	0	1	0	1	0	1
Quality	12	9	11	12	13	13	12	11	9	7	14	12	13

Article

[9]. Elchert J, Beaudrot M, DeFranco E. Gestational Weight Gain in Adolescent Compared with Adult Pregnancies: An Age-Specific Body Mass Index Approach. *J Pediatr*. **2015**; 167(3):579-85.e1-2. https://doi.org/10.1016/j.jpeds.2015.05.043

[32]. Cunningham SD, Mokshagundam S, Chai H, Lewis JB, Levine J, Tobin JN, et al. Postpartum Depressive Symptoms: Gestational Weight Gain as a Risk Factor for Adolescents Who Are

Overweight or Obese. *J Midwifery Women's Health*. **2018**;63(2):178-84. https://doi.org/10.1111/jmwh.12686

[33]. Danilack VA, Brousseau EC, Phipps MG. The Effect of Gestational Weight Gain on Persistent Increase in Body Mass Index in Adolescents: A Longitudinal Study. *J Women's Health (Larchmt)*. **2018**;27(12):1456-8. https://doi.org/10.1089/jwh.2017.6635

[34]. Groth SW. The long-term impact of adolescent gestational weight gain. *Res Nurs Health*. **2008**;31(2):108-18. https://doi.org/10.1002/nur.20243

[35]. Ekambaram M, Irigoyen M, DeFreitas J, Rajbhandari S, Geaney JL, Braitman LE. Gestational weight gain among minority adolescents predicts term birth weight. *World J Pediatr*. **2018**;14(4):357-63. https://doi.org/10.1007/s12519-018-0143-9

[36]. Joseph NP, Hunkali KB, Wilson B, Morgan E, Cross M, Freund KM. Pre-pregnancy body mass index among pregnant adolescents: gestational weight gain and long-term postpartum weight retention. *J Pediatr Adolesc Gynecol*. **2008**;21(4):195-200. https://doi.org/10.1016/j.jpag.2007.08.006.

[43]. Chu SY, Callaghan WM, Bish CL, D'Angelo D. Gestational weight gain by body mass index among US women delivering live births, 2004-2005: fueling future obesity. *Am J Obstet Gynecol*. **2009**;200(3):271.e1-7. https://doi.org/10.1016/j.ajog.2008.09.879.

[37]. Timur H, Kokanalı MK, Topçu HO, Topçu S, Erkılınç S, Uygur D, et al. Factors That Affect Perinatal Outcomes of the Second Pregnancy of Adolescents. *J Pediatr Adolesc Gynecol*. **2016**;29(1):18-21. https://doi.org/10.1016/j.jpag.2015.05.002.

[38]. Groth SW, Holland ML, Smith JA, Meng Y, Kitzman H. Effect of Gestational Weight Gain and Prepregnancy Body Mass Index in Adolescent Mothers on Weight and Body Mass Index of Adolescent Offspring. *J Adolesc Health*. **2017**;61(5):626-33. https://doi.org/10.1016/j.jadohealth.2017.05.005

[39]. Noreña I, Pardo MP, Mockus I. Serum adipokine levels and insulin resistance in the first trimester of pregnancy in adolescents and their relationship with neonatal weight. *Biomedica*. **2018**;38(3):427-36. https://doi.org/10.7705/biomedica.v38i4.4035

[40]. Sámano R, Martínez-Rojano H, Chico-Barba G, Godinez-Martínez E, Sánchez-Jiménez B, Montiel-Ojeda D, et al. Serum Concentration of Leptin in Pregnant Adolescents Correlated with Gestational Weight Gain, Postpartum Weight Retention and Newborn Weight/Length. *Nutrients*. **2017**;9(10). https://doi.org/10.3390/nu9101067.

[41]. Sam-Soto S, Sámano R, Flores-Ramos M, Rodríguez-Bosch M, García-Salazar D, Hernández-Mohar G, et al. Ganancia de peso durante la gestación y resultados perinatales en adolescentes embarazadas con antecedente de abuso sexual. *Nutrición Hospitalaria*. **2015**;32(3):1075-81. http://dx.doi.org/10.3305/nh.2015.32.3.9377.

[42]. Sámano R, Martínez-Rojano H, Chico-Barba G, Sánchez-Jiménez B, Illescas-Zarate D, Rodríguez-Ventura AL. Characteristics of the Family Support Network of Pregnant Adolescents and Its Association with Gestational Weight Gain and Birth Weight of Newborns. *Int J Environ Res Public Health*. **2019**;16(7). https://doi.org/10.3390/ijerph16071222.
